# Canadian Emergency DepartmentTriage and Acuity Scale: implementation in a tertiary care center in Saudi Arabia

**DOI:** 10.1186/1471-227X-11-3

**Published:** 2011-02-10

**Authors:** Naser B Elkum, CarolAnne Barrett, Hisham Al-Omran

**Affiliations:** 1 Department of Emergency Medicine, King Faisal Specialist Hospital and Research Center, PO Box 3354, Riyadh 11211, Saudi Arabia; 2 Department of Biostatistics & Epidemiology, Dasman Diabetes Institute, PO Box 1180, Dasman 15462, Kuwait; 3 Department of Biostatistics, Epidemiology and Scientific Computing, King Faisal Specialist Hospital and Research Center, PO Box 3354, Riyadh 11211, Saudi Arabia

## Abstract

**Background:**

The Canadian Emergency Department Triage and Acuity Scale (CTAS) is a well recognized and validated triage system that prioritizes patient care by severity of illness. The aim of this study was to describe the results of Emergency Department (ED) waiting times after the implementation of the CTAS in a major tertiary care hospital emergency department outside of Canada.

**Methods:**

A total of 1206 charts were randomly selected and retrospectively reviewed for triage performance. The indicators were: time to triage, triage duration, waiting time to be evaluated by a physician, and proportion of patients who left without being seen by a physician. Waiting times were stratified by triage level and reported as fractile response rates.

**Results:**

The approximate time to triage was ≤ 10 minutes for 71% and ≤ 15 minutes for 82.8% of the patients. Fifty-three percent (53.5%) completed their triage process within 5 minutes. Waiting times evaluated by a physician was 100% within CTAS time objectives in category I patients, however, this was not the case for the other 4 categories. The overall left without being seen (LWBS) rate was 9.8%; 11.9% were in Level III, 20.3% in Level IV, and 67.8% in Level V. Median length of stay (LOS) was 144 minutes for the study sample as a whole.

**Conclusion:**

The CTAS may be adapted, with achievable objectives, in hospitals outside Canada as well. Time to see physician, total LOS, and LWBS are effective markers of ED performance and the quality of triage. Registration-to-physician time (RTP) and LOS profiles, stratified by triage level, are essential indicative markers for ED performance and should be used in improving patients flow through collaborative efforts.

## Background

Emergency care is typically sought for serious injuries and acute medical conditions (i.e. heart attack or stroke), however, excessive delays and overcrowding of emergency departments (EDs) have become serious problems, thus, causing concern with regards to compromise in care. Accordingly, longer waiting times in the ED not only contribute to patients' dissatisfaction with the care received [1], but may also result in delays in diagnosis and treatment [2,3], as well as, chronic pain and suffering. In addition, a large segment of patients bombard the ED with lesser acute complaints, sometimes preoccupying medical staff time and resources, and delaying the management of more acutely ill patients [4-7].

An ideal triage system should prioritize patient care by severity, and that care should be delivered within a reasonable time frame. A well recognized and validated triage system is the Canadian Emergency Department Triage and Acuity Scale (CTAS) [8]. CTAS has five acuity levels to V consisting of - Resuscitation, Emergent, Urgent, Less Urgent and Non Urgent. The CTAS accurately defines patients' acuity level, which assists ED staff members to better evaluate patients, department resources needs, and performance against certain operating objectives. Literature of studies that validate CTAS outside the Canadian healthcare system is scarce [9].

King Faisal Specialist Hospital & Research Center is a major tertiary care institution, serving patients referred throughout the Kingdom of Saudi Arabia and Middle East, and hence, the expectations of these patients are very high. The ED is an important entry point to the health care system in the institution. The ED is a 30 bed unit based within an 800 bed tertiary care center. The ED serves all critical patients and those patients followed up at the various sub-specialty departments. It has an annual volume of 65,000 patients, with 73% of them being above 14 years of age. A large percentage of the patients are followed up for tertiary care problems in several specialties, such as oncology including bone marrow transplant, cardiovascular diseases, neurosciences, medical genetics, and renal and liver transplants. Since the hospital functions as a highly specialized central tertiary care center for the country, the patient mix is quite different than other general hospitals. Our hospital receives patients with tertiary care needs from a large geographic area, as these individuals do not have access to tertiary care elsewhere in the country. Prolonged waiting before treatment in the ED may negatively color patients' perceptions about their care providers during such visits. The need for the use of an objective process of patient prioritization, and the theoretical applicability of the CTAS to any ED, prompted us to implement the CTAS system in the institution.

The CTAS has been extensively studied and validated in a variety of settings [9-11]; however, these studies were done in areas where large integrated health care systems are already established, unlike in Saudi Arabia where patients do not necessarily have an identifiable primary care provider. Additionally, our patient population has unique cultural and linguistic features that are not present in other studies. Our study is the first in an Arab country that aims to evaluate the feasibility and validity of CTAS by comparing certain ED quality indicators with pre-established CTAS triage objectives, and to evaluate the relationship between CTAS triage level and waiting times.

## Methods

This retrospective study was performed using randomly selected patients who presented to the Emergency Department of the King Faisal Specialist Hospital and Research Center, between November 2004 and February 2005. The study was approved by the Institutional Review Board (Research Ethics Board) of King Faisal Specialist Hospital and Research Center.

### Data Collection

A random sample of 25 charts was selected every day for the 4 month study period. The registration clerk, triage nurse and evaluating physician recorded ED patient's arrival time, triage time and time seen by physician respectively, on the patient's chart during his/her visit. The CTAS was used to assign triage level by the triage nurse upon initial assessment, and the following variables were collected: day of arrival, demographic data, triage level, time at triage, room assignment time, time seen by ED physician, the time of disposition, admission or discharge. Time of arrival/registration was defined as the time when the patient approached the ED registration desk to express his or her desire to be treated.

### Time Intervals

Time intervals for ED assessment and treatment were calculated, and total length of stay (LOS) was determined for each patient. Time intervals were defined as: 1) time to triage assessment (TTA)- the time from registration until initiation of triage, 2) triage duration (TD)- the total time for the nurse to complete triage assessment, 3) registration to physician time (RPT)- the patient waiting time from initial registration until evaluation by the ED physician, 4) time from physician assessment to final disposition decision (TPD), and finally, 5) total length of stay (LOS)- the time from patient registration to final disposition made by the ED physician. Fractile response rates were used to describe RPT and LOS. Fractile response rates specify the proportion of patients in each triage level seen within the CTAS time objective for that level [8,12].

### Quality Indicators

Four quality parameters were set to assess ED performance using the CTAS guidelines: 1) TTA, which should be ≤ 10 minutes; 2) TD, which should be ≤ 5 minutes; 3) RPT, which should be less than 5 minutes, 15 minutes, 30 minutes, 60 minutes, and 120 minutes for CTAS categories I, II, III, IV, and V respectively; and 4) proportion of patients leaving without being seen (LWBS) by a physician, which should be < 2%.

### Data Analysis

A Palm Pilot personal digital assistant (PDA) data-entry system was used to input the ED patient's information. The data was downloaded directly from the PDA into a Microsoft Access database for further analysis. This is the first research study using a PDA in this hospital. Statistical analysis was performed using SAS^® ^software (version 9.1.2; SAS institute, Cary, NC). We used standard descriptive statistics including medians, means and standard deviations to characterize the sample of patients and waiting times.

## Results

During the study period, 1206 charts were randomly selected from the medical records of patients who were triaged in our ED. This number represents the charts that were available at the medical records department at time of selection. The mean age of study patients was 29.9 years (standard deviation (SD) = 23.0 years). Of the total patients, 32.6% were less than 14 years old. Approximately, half (52.5%) of the study population were females. The distribution of patients per triage category were: Level I (0.2%), Level II (0.4%), Level III (23.6%), Level IV (59.6%) and 16.1% in Level V (Table 1).

**Table 1:** Key emergency department process intervals (median minutes), stratified by triage level.

Triage level	n (%)	Registration to triage	Triage to physician	Physician to disposition decision	Total ED LOS (SD)
I	2 (0.17)	1.0	2.0	303.0	373.5 (94.05)
II	5 (0.41)	5.0	0.0	277.5	329.0 (145.04)
III	284 (23.55)	6.0	35.0	185.0	246.0 (259.1)
IV	721 (59.78)	6.0	45.0	40.0	130.0 (157.7)
V	194 (16.09)	8.0	30.0	20.0	88.0 (122.7)
All	1206 (100)	6.0	40.0	50	144.0 (192.3)

**Figure 1:**
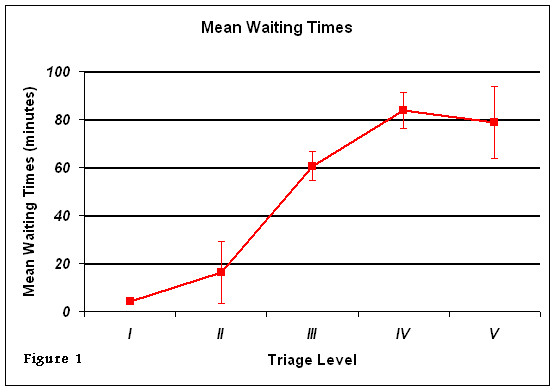
**Mean waiting time from registration to being seen by physician in the emergency department by triage category**.

The median waiting time from registration to being seen by a physician, (RTP), was 53.0 min (range 0.0 min to 1330.0 min). As expected, RTP varied by triage category (Figure 1) with level IV and V patients having the longest RTP times. TTA was ≤ 10 minutes for 854 patients (70.8%) and ≤ 15 minutes for 998 (82.8%). Triage duration was less than 5 minutes for 645 patients (53.5%). Table 2 shows the mean RTP time and fractile response rates for each CTAS level. All category I patients met the set CTAS standard, however, this was not so in the other 4 categories.

**Table 2:** Comparison of physician response times in study with CTAS response time objectives.

Triage level	Mean time from registration to physician assessment		Physician assessment *fractile response, %
		
	Study, min (SD)	CTAS objective min	
I	4 (-)	5	100
II	16.3 (13.2)	15	60
III	60.5 (48.9)	30	36
IV	83.7 (92.9)	60	61
V	78.9 (50.0)	120	83

*Fractile response defines the percentage of patients seen within specified CTAS time objectives for each triage level.

**Figure 2:**
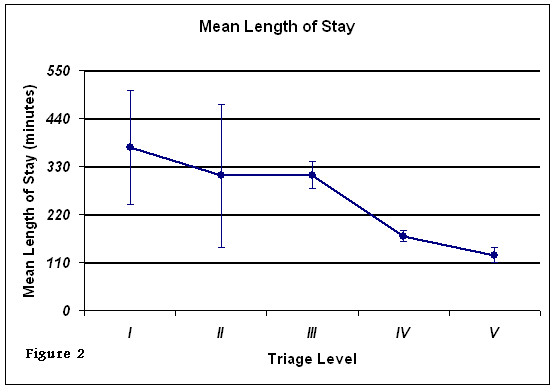
**Mean length of stay in the emergency department by triage category**.

In our study sample, 81 patients (6.7%) were hospitalized, 118 patients (9.8%) LWBS and 1007 (83.5%) were discharged. Of the 118 patients who LWBS, 11.9% were Level III, 20.3% were Level IV and 67.8% were Level V. The median time these patients waited before leaving was 133.0 minutes (95% CI, 119.9 - 153.2 min). The median ED LOS was 144 minutes for the study sample as a whole. Figure 2 shows an increase in LOS with triage acuity.

## Discussion

Our data assessed triage performance, timeliness of care, and length of stay in ED. It evaluated the feasibility and validity of the CTAS outside of Canada. Our results show an indirect relationship between CTAS acuity level and RTP: as CTAS acuity level increased, RTP decreased and LOS increased. ED patients who left the ED without being seen were of low CTAS acuity levels.

The majority of our ED patients were category IV and V (75.7%), which is in line with the percentage of CTAS IV and V in the Principality of Andorra ED of 76.82% [9]. The lower percentage of levels I & II (0.6%) could be due to many reasons such as random errors, or assigning a patient an inappropriate low triage level. This is not a trauma hospital and, hence, this could be another reason leading to low percentages of levels I & II. Tables 1 and 2 demonstrate the RTP time generally increased as triage acuity fell. Although this is expected, fractile response rates were actually higher in levels IV and V (61% and 83% respectively), than in level III (36%). This lower fractile response rate could be due to a variety of reasons including space limitations, eligibility for care at this hospital, ED volume, or language spoken. Bias and prejudice might also play a role in this lower response rate.

Our data also showed that, for the most critically ill patients (level I&II), RTP was rapid and LOS was greatest, which are in line with CTAS objectives. This is expected because these patients required more time and manpower resources for the care and management of their critical condition, thus, contributing to a prolonged LOS in the ED (Figure 2 and Table 1).

Hospitalization rate is a marker of the severity of illness. Hospital admission rate through our ED, in this study, was 6.7%, which is in agreement with other studies [9,13]. However, other studies showed higher percentage of hospitalization through the ED [7,14]. These variations in hospital admission rates could be due to several factors including hospital size, number and types of specialties in the hospital, triage system, patients' eligibility, and insurance coverage. Admission rates are generally correlated with CTAS triage level; in this study, the majority of our ED patients were categorized as levels IV and V. Furthermore, our hospital is a specialized tertiary care institute, where patients are transferred from other hospitals in the region. This may explain, in part, the low admission rates through the ED.

Previous studies showed that up to 15% of patients left ED without receiving any medical attention [15-18]. Likewise, our ED's estimated LWBS rate is approximately 9.8%, however, this is higher than our quality indicator of < 2%. Using CTAS, recent study in United Arab Emirates, showed a rate of 4.7% LWBS [19], Canadian studies reported rates between 3 - 3.57% [20,21], and 7.4 - 15.0% in the USA [17,22-24]. These international variations in LWBS may reflect differences in culture, ED structure or service delivery. "Left without being seen" is related to many factors, such as ED efficiency, patient volume and acuity, understaffing and overcrowding [23,25]. In keeping with CTAS objectives, our data demonstrated that of 118 patients, who left without being seen during the study period, none were in Levels I or II (Resuscitation or Emergent), and only 14 (11.9%) were in Level III. This implies that in our ED patients who LWBS, generally, have conditions of a less acute and less urgent nature.

Waiting time studies offer constructive information to identify system inefficiencies and for benchmarking purposes. With a growing population and an increasing demand for medical care in EDs throughout the Gulf region and elsewhere, there is a need for comparative studies both locally, as well as, internationally to document and account for avoidable areas of delay in the care of emergency patients, and hence, improve quality of care. Our study is one of a few, which examines the CTAS in EDs outside of Canada.

## Limitations

The data presented in this study comes from only one institution, which may limit the ability to generalize our results to other facilities, because this institute has different setting and patient characteristics, than most of the CTAS published studies. However, we believe that the outcomes reflect the reality of most EDs that use CTAS.

## Conclusion

We conclude that the CTAS may be implemented, with achievable objectives, in hospitals outside Canada. Time to see physician, total LOS, and LWBS are effective markers of performance of ED and the quality of triage. RTP and LOS profiles, stratified by triage level, are essential for the management of ED and improving patient flow through collaborative efforts.

## Competing interests

The authors declare that they have no competing interests.

## Authors' contributions

NE conceived and designed the study, data acquisition, data analysis and interpretation, and wrote the manuscript. CB and HA participated in the critical revision of the manuscript. All authors have read and approved the final version of the manuscript.

## Pre-publication history

The pre-publication history for this paper can be accessed here:

http://www.biomedcentral.com/1471-227X/11/3/prepub
